# The 20th Anniversary Meeting of the Rocky Mountain Virology Association

**DOI:** 10.3390/v13010038

**Published:** 2020-12-29

**Authors:** Joel Rovnak, Laura A. St Clair, Carley McAlister, Chinemerem P. Ogbu, Jessica Smolenske, Randall J. Cohrs, Rushika Perera

**Affiliations:** 1 Department of Microbiology, Immunology and Pathology, Colorado State University, Fort Collins, CO 80523, USA; Joel.Rovnak@colostate.edu; 2 Center for Vector-Borne Infectious Diseases, Department of Microbiology, Immunology and Pathology, Colorado State University, Fort Collins, CO 80523, USA; stclairl@colostate.edu (L.A.S.C.); carleym@rams.colostate.edu (C.M.); rushika.perera@colostate.edu (R.P.); 3 Department of Biochemistry and Nebraska Center for Virology, University of Nebraska-Lincoln, Lincoln, NE 68583, USA; cogbu2@huskers.unl.edu; 4 Mountain Campus Program Support, Colorado State University, Fort Collins, CO 80523, USA; Jessica.Smolenske@colostate.edu; 5 Departments of Neurology and Immunology/Microbiology, University of Colorado School of Medicine, Aurora, CO 80045, USA

**Keywords:** coronavirus, virus, prion, immune suppression, flavivirus, retrovirus, poxviruses, host-virus interaction, herpesvirus

## Abstract

Due to the COVID-19 pandemic and multiple devastating forest fires, the 2020 meeting of the Rocky Mountain Virology Association was held virtually. The three-day gathering featured talks describing recent advances in virology and prion research. The keynote presentation described the measles virus paradox of immune suppression and life-long immunity. Special invited speakers presented information concerning visualizing antiviral effector cell biology in mucosal tissues, uncovering the T-cell tropism of Epstein-Barr virus type 2, a history and current survey of coronavirus spike proteins, a summary of Zika virus vaccination and immunity, the innate immune response to flavivirus infections, a discussion concerning prion disease as it relates to multiple system atrophy, and clues for discussing virology with the non-virologist. On behalf of the Rocky Mountain Virology Association, this report summarizes selected presentations.

## 1. Introduction

**Figure 1:**
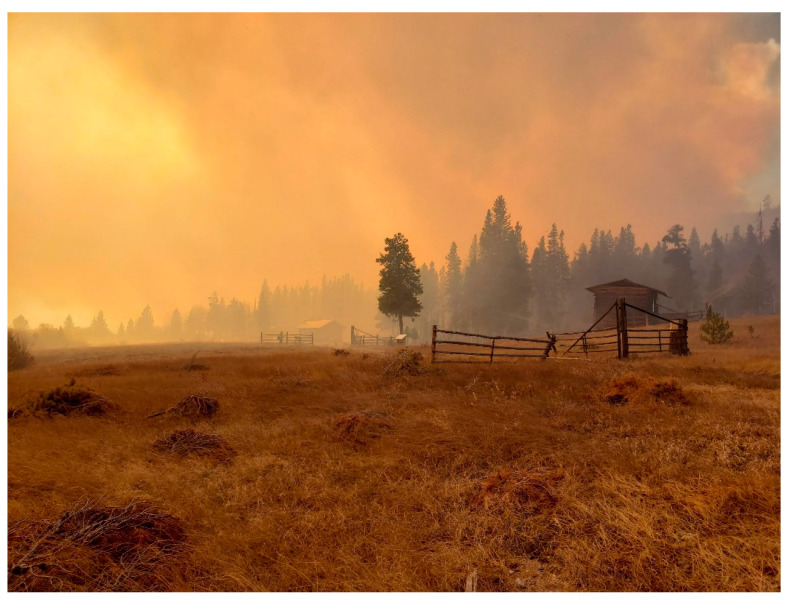
Smoke and ash from the Cameron Peaks fire filled the valley and surrounded the historic Koenig-Ramsey Homestead District at Colorado State University. The heroic efforts of several fire crews successfully protected the >100 years old structures.

**Figure 2:**
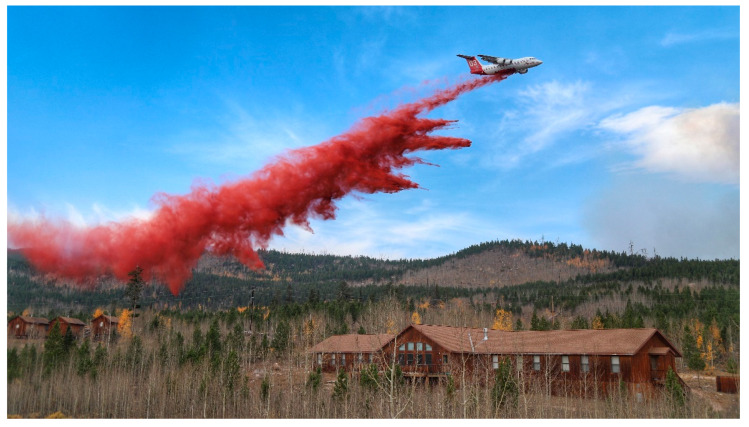
An aerial suppression plane dropped fire retardant along the fire-line just west of the Colorado State University Mountain Campus north dorm. Although the flames crossed the main fire-line, suppression efforts were successful and protected the residential and guest cabins.

In 2000, the Rocky Mountain Virology Club was formed to showcase regional scientists investigating all facets of virus/host interactions, to provide an environment where graduate students could interact with more established virologists, and new investigators could initiate productive collaborations. In 2009, Prions were added to the Club and in 2013 the Club became an Association. Over the decades, attendance has grown from a few dozen local participants to over 80 attendees with invited speakers both national and international. In October 2020 we celebrated our 20th anniversary, not at our traditional venue secluded within the center of the Rocky Mountains, but at our individual computers in a virtual meeting that was due ironically to the global coronavirus pandemic. In addition, the summer of 2020 saw the largest forest fires in Colorado’s history. Our venue, the Colorado State University Mountain Campus, functioned as the base for firefighting activity until the inferno encroached within the campus boundary (Figure 1). However, combined ground and aerial assaults (Figure 2) saved the campus where next year’s meeting is planned.

This year’s three-day meeting was attended by at least 123 individuals who experienced 36 presentations including Dr. Diane Griffin who presented the keynote talk and invited lectures by Drs. Margo A. Brinton, Amanda L. Woerman, Heather Hickman, David Quammen, Rosemary Rochford, Kathryn Holmes, and Theodore Pierson. Our professional childcare director organized the kids virtually for three comical interludes where the kids’ skits were COVID-based. Along with the presentations covering current findings in virology and prion biology, David Quammen, science journalist and author of *Spillover* discussed his methods for conveying virology to the non-virologist. He reminded us that science is a human process, and readers enjoy reading human interest stories. He also relayed that the best way to relay science to public audiences is to first go to the source and talk to the people involved in the research. Finally, he reminded us that it is important to remember the difference between accuracy and precision, how best to convey those meanings to public audiences, and the power of metaphors to convey complex scientific topics. Taken together, the 20th annual RMVA virtual meeting successfully united an international group of students and virologists in discussions that covered RNA and DNA viruses along with recent advances in prion biology. Selected abstracts are presented below.

## 2. Summary of Scientific Sessions

### 2.1. Keynote Speaker

Diane E. Griffin (W. Harry Feinstone Department of Molecular Microbiology and Immunology, Johns Hopkins Bloomberg School of Public Health, Baltimore, MD, USA) discussed the paradox of immune suppression and life-long immunity induced by measles virus infection. The effects of measles virus on the immune system are only partially understood. Lymphoid tissue is a primary site of measles virus (MeV) replication where CD150 is the receptor for infection of both B and T cells. Lymphocyte depletion occurs during the acute phase of infection, but initiation of the adaptive immune response leads to extensive lymphocyte proliferation, production of MeV-specific antibody and T cells, the rash and clearance of infectious virus. Viral RNA persists in lymphoid tissue for months accompanied by ongoing germinal center proliferation, production of antibody-secreting cells, functionally distinct T cells and antibody avidity maturation to establish life-long immunity. The live virus vaccine replicates less well in lymphocytes and induces a less robust and less durable antibody response. During wild type measles, but not vaccine infection, diversity of pre-existing antibodies and numbers of memory and naive B cells are reduced and susceptibility to other infections is increased. Funding for this work was provided by the National Institute of Health, grant numbers R01AI131228, R21AI095981, R01AI153140. All animal studies were performed following guidelines and protocols approved by the Institutional Animal Care and Use Committee of Johns Hopkins University.

### 2.2. Session I

Benjamin Akiyama along with Monica E. Graham^2^, Zoe O’Donoghue^1^, J. David Beckham^3^, and Jeffrey S. Kieft^1,4^ (^1^Department of Biochemistry and Molecular Genetics, ^2^Department of Immunology and Microbiology, ^3^Department of Medicine Division of Infectious Diseases, and ^4^RNA BioScience Initiative, University of Colorado Denver School of Medicine, Aurora, CO, USA) presented their research on how the three-dimensional structure of a flavivirus dumbbell RNA reveals molecular details of an RNA regulator of replication. Mosquito-borne flaviviruses (MBFVs) including dengue, West Nile, yellow fever, and Zika viruses have an RNA genome encoding one open reading frame with 5′ and 3′ untranslated regions (UTRs). The 3′ UTRs of MBFVs contain regions of high sequence conservation in elements known as dumbbells (DBs) that regulate translation and replication of the viral RNA genome, functions proposed to depend on the formation of an RNA pseudoknot. To understand how DB structure provides this function, they used crystallography and structural modelling to reveal its tertiary structure. The crystal structure confirmed the predicted pseudoknot and molecular modeling revealed how conserved sequences form a four-way junction that stabilizes the pseudoknot. Single-molecule FRET suggests that the DB pseudoknot is a stable element that can regulate the switch between translation and replication during the viral lifecycle by sequestering replication signals. No animal or human studies were performed.

Margo Brinton (Department of Biology, Georgia State University, Atlanta, GA, USA) presented her research on the RNase L-dependent and RNase L-independent anti-flavivirus activities of cellular 2′–5′ oligoadenylate synthetase 1 (OAS 1) proteins. OAS proteins are activated by double-stranded regions in RNA and use ATP to synthesize short A oligos with a 2′–5′ linkage. The 2′–5′ A binds to the constitutively expressed cytoplasmic endonuclease RNase L inducing it to dimerize and cleave viral and cellular single stranded RNAs at UA and UU dinucleotides. OAS gene expression is upregulated in West Nile virus (WNV) infected cells by interferon signaling. WNV genomic RNA is susceptible to RNase L cleavage and virus yields are ~10 fold higher in cells without RNase L. The human genome encodes one OAS1 gene while the mouse genome encodes 8 OAS1 genes, but only two of the mouse OAS1 proteins, OAS1a and OAS1g, are active synthetases. The full-length mouse OAS1b protein confers an RNase L-independent antiviral activity that is specific for members of the genus Flavivirus. Virus attachment, entry and initial viral RNA replication levels are equivalent in resistant and susceptible (express a C-terminally truncated protein) cells but subsequent amplification of nascent genome RNA synthesis is inhibited in resistant cells. Resistant mice show no symptoms and survive after an intracranial injection of a virulent flavivirus that kills 100% of susceptible mice. Even though evidence indicated that activated RNase L reduced the levels of OAS1b mRNA in the brains of WNV-infected resistant mice, the OAS1b-mediated resistance phenotype was robust. Several lines of research are in progress to investigate the mechanism of action of the OAS1b resistance. Two Oas1b cellular interaction partners, ABCF3 and ORP1L, were identified. The ATPase activity of ABCF3 was enhanced by the binding of a subset of lipid ligands similar to types of lipids upregulated in flavivirus-infected cells. OAS1b also has ATPase activity, but a synergistic increase in ATPase activity was not observed for the ABCF3-OAS1b complex. Whether ATPase activity plays a role in the resistance phenotype is not yet known. Funding for this project was provided by: NIAID, National Institutes of Health, R01 AI45135. All animal studies were performed following guidelines and protocols approved by the Institutional Animal Care and Use Committee of Georgia State University.

Jaquelin P. Dudley^1,2^ along with Hyewon Byun^1^, Gurvani Singh^1^, Brynn Weakley^1^, Jazmin Urrutia^1^, and Mary M. Lozano^1^ (^1^Department of Molecular Biosciences, ^2^LaMontagne Center for Infectious Disease, and Institute for Cellular and Molecular Biology, The University of Texas at Austin, Austin, TX, USA) presented their work uncovering the acceleration of retrovirus-induced lymphomas in the absence of a viral Apobec antagonist. Retroviruses have evolved multiple mechanisms for immune evasion. Mouse mammary tumor virus (MMTV) is a complex retrovirus that is antagonized by murine Apobec3 (mA3), a member of the Apobec family of cytidine deaminases involved in innate and adaptive immunity. MMTV requires replication in both B and T cells for viral transmission. They recently showed that MMTV-encoded Rem protein antagonizes proviral mutagenesis by one or more Apobec enzymes, including the activation-induced cytidine deaminase (AID), in BALB/c mice. To further study the role of Rem in vivo, they used TBLV, a superantigen-independent T-lymphomagenic strain of MMTV, to infect C57BL/6 (B6) mice. At high viral doses, infections of B6 mice with Rem-mutant TBLV (TBLV-SD) gave a slightly shorter latency, but lower proviral loads relative to wild-type TBLV (TBLV-WT). The shortened latency of TBLV-SD-induced tumors was abolished in *Aicda*^−/−^ mice. Mutational differences between TBLV-WT and the Rem-null mutant proviruses from B6 T-cell lymphomas were reduced compared to those detected in BALB/c mice. Consistent with TBLV replication primarily in T cells, mutations typical of mA3 were the most affected by the absence of Rem expression. Surprisingly, at lower viral doses in B6 mice, they observed markedly accelerated T-cell tumor induction by the Rem-mutant virus compared to TBLV-WT, but little difference in proviral loads. To explain the shortened latency of T-cell tumors by the Rem-mutant, they are currently using RNASeq. Their results suggest that multiple Apobec enzymes are antagonized by Rem during MMTV and TBLV replication in mice. Furthermore, Apobec-mediated mutagenesis may select for viruses with enhanced oncogenicity. Funding for this work was provided by the National Institute of Health, grant numbers R01CA167053 and R01AI131660. All animal studies were performed following guidelines and protocols approved by the Institutional Animal Care and Use Committee of The University of Texas at Austin.

Monica E. Graham^1^ along with Akiyama BM^2^ and Beckham JD^1^ (^1^Department of Immunology and Microbiology, ^2^Department of Biochemistry and Molecular Genetics, University of Colorado Anschutz Medical Campus, Aurora, CO, USA) presented their work on the structure–function relationship of the Zika virus DB-1 structure during viral infection. They showed Zika virus (ZIKV) contains multiple conserved RNA structures and sequences in the viral 5′ and 3′ untranslated regions (UTRs). The dumbbell-1 structure (DB-1) is one of the least studied RNA structures in the 3′UTR. Previous research in other flaviviruses has demonstrated that the DB-1 structure in Zika virus and other flaviviruses is important for viral genome replication and cytopathic effect of the virus. However, these studies have not investigated how the highly conserved structure of DB-1 is related to its function, and few studies have been done in ZIKV. They created two mutant, infectious ZIKV clones: TL.PK, which disrupts a predicted tertiary fold of DB-1; and p.2.5′, which disrupts predicted conserved secondary structure. For both mutants, viral genome replication is modestly abrogated in mammalian cell cultures, and no different from wild type in mosquito culture. Their TL.PK mutant produced comparable levels of infectious virions to wild type in both mammalian and mosquito cells. However, the p.2.5′ mutant produced significantly less infectious virions in both mammalian and mosquito cells culture. For both mutants, cytopathic effect is visibly attenuated, with p.2.5′ being more attenuated than the TL.PK mutant. These data suggest that the DB-1 structure plays an important role in viral pathogenesis, and that the conserved secondary structure of DB-1 is likely more relevant to proper function than the predicted tertiary structure. The funding for this project comes from the Department of Defense and the Department of Veterans Affairs (DOD MIIRA PRMRP PR160117, VA Merit I01 BX003863). No animal or human studies were performed.

Alexandria C. Linville^1,3^, along with Rico AB^2,3^, Olson AO^1,3^, and Wiebe MS^2,3^ (School of Biological Sciences, University of Nebraska-Lincoln^1^, School of Veterinary Medicine and Biomedical Sciences, University of Nebraska-Lincoln^2^, and Nebraska Center for Virology, University of Nebraska-Lincoln^3^, Lincoln, NE, USA) presented their work on how Vaccinia pseudokinase inhibits viral replication by activating the host restriction factor BAF. Poxviruses are unique among mammalian dsDNA viruses in their ability to replicate in the cytoplasm of the cell. The complex signaling pathways that vaccinia virus engages to modulate host defense responses and establish a successful viral life cycle are only partially understood. It is known that the viral B1 kinase plays an important role in this modulation by phosphorylating and inactivating the cellular anti-viral protein BAF. In searching for other key roles B1 may play, it was discovered that an attenuated vaccinia virus lacking the B1 gene exhibits enhanced fitness after mutating and ablating the function of the viral B12 protein. Furthermore, decreased activity of the B12 protein has been correlated with influencing the phosphorylation state of cellular BAF. Their recent studies of the B12 interactome have discovered a complex comprised of B12 and cellular vaccinia related kinase 1 (VRK1), a cellular homolog of B1 and B12. From these key observations, they hypothesized that this viral B12 pseudokinase/cellular VRK1 kinase complex influences VRK1 signal transduction and facilitates B12′s repressive activity against vaccinia virus. This theory has been supported by studies characterizing the fitness of a panel of vaccinia viruses containing B1-deletions and/or B12 mutations in VRK1 depletion and VRK1 over-expression conditions. Interestingly, parallel studies also implicate the B12/VRK1 complex in influencing the phosphorylation state of BAF. In summary, these insights into the influence of the B12/VRK1 complex on vaccinia virus fitness have yielded important knowledge regarding the mechanism of a novel nuclear remodeling pathway that occurs during poxvirus infection. This research was supported through National Institutes of Health grants: R01AI114653 and T32AI125207. No animal or human studies were performed.

Hadrian Sparks^1^, along with Brendan Monogue^1^, Benjamin M. Akiyama^3^, Jeffrey S. Kieft^3,4^, and J. David Beckham^1,2^, (^1^Department of Immunology & Microbiology, ^2^Department of Medicine, Division of Infectious Diseases University of Colorado School of Medicine, ^3^Department of Biochemistry and Molecular Genetics and ^4^RNA BioScience Initiative, University of Colorado Anschutz Medical Campus, Aurora, CO, USA) presented their work on the Disruption of Zika virus xrRNA1-dependent sfRNA1 production results in tissue-specific attenuated viral replication. They showed that similar to other flaviviruses, Zika virus (ZIKV), produces several species of sub-genomic RNAs (sfRNAs) during infection, which are noncoding RNA fragments of different lengths derived from the viral 3′ untranslated region (UTR). During infection, these sfRNAs accumulate in the host cell as a result of incomplete viral genome degradation of the 3′UTR by the host 5′ to 3′ exoribonuclease (Xrn1). The stopping of Xrn1 at the 3′UTR is due to two RNA tertiary structures in the 3′UTR termed exoribonuclease-resistant RNA1 and 2 (xrRNA1&2). Investigations with similar flaviviruses have shown that sfRNAs are important for pathogenicity and inhibiting both mosquito and mammalian host defense mechanisms. However, these investigations have largely excluded ZIKV. Additionally, there are very limited data addressing how sfRNAs impact infection in a whole animal model or specific tissues. They have rescued an infectious sfRNA1-deficient ZIKV (X1) by targeted mutation in the xrRNA1 3′ UTR structure. They found the X1 virus lacks the production of the largest ZIKV sfRNA species, sfRNA1. Using the X1 virus to infect adult *IFNAR1^−/−^* mice, they found that while the lack of sfRNA1 does not alter ZIKV replication in the spleen, there is a significant reduction in ZIKV genome replication in the brain and placenta compared to WT ZIKV infection. Despite the attenuated phenotype of the X1 ZIKV, mice develop a robust neutralizing antibody response. They conclude that targeted disruption of xrRNA1 results in tissue-specific attenuation while still supporting robust neutralizing antibody responses. Future studies will investigate the tissue-specific mechanisms by which ZIKV sfRNAs influence infection and may utilize targeted xrRNA mutations to develop novel attenuated flavivirus vaccine approaches. This work was supported by DOD PRMRP funding (contract W81XWH-17-1-0183) and VA Merit Funding (I01BX003863) to J.D.B. All animal studies were performed following guidelines and protocols approved by the Institutional Animal Care and Use Committee of the University of Colorado Anschutz Medical Campus.

### 2.3. Session II

Stephanie E. Ander, along with Kathryn S. Carpentier, and Thomas E. Morrison (Department of Immunology and Microbiology, University of Colorado School of Medicine, Aurora, CO, USA), presented their talk on defining the molecular determinants of encephalitis alphavirus viremia. The magnitude and duration of vertebrate viremia is a critical determinant of arbovirus transmission, geographic spread, and disease severity. However, the mechanisms that determine arboviral viremia levels are poorly defined. Previously, they found that multiple arthritogenic alphaviruses, including chikungunya virus (CHIKV), are cleared from the circulation of mice by phagocytic cells. Clearance of these viruses from murine circulation is efficient, independent of natural antibodies or complement factor C3, and relies on the presence of scavenger receptor SR-A6 (MARCO) and Kupffer cells (liver macrophages). To determine whether this clearance pathway is specific for arthritogenic alphaviruses or functions more broadly, they evaluated the clearance of circulating encephalitic alphaviruses, including Eastern- (EEEV) and Venezuelan- (VEEV) equine encephalitis viruses using recombinant virus particles composed of a chimeric SINV-EEEV^FL93^, -VEEV^TrD^, or -VEEV^PIXV^ genome encapsulated by EEEV^FL93^, VEEV^TrD^, or VEEV^PIXV^ structural proteins. Following intravenous inoculation, they found that EEEV^FL93^ and VEEV^PIXV^ particles were cleared from murine circulation while VEEV^TrD^ particles were remarkably resistant to clearance; persisting within the bloodstream beyond 3 hpi. Congruent with their earlier studies with CHIKV, (1) depletion of phagocytic cells by clodronate-loaded liposomes abrogated clearance of circulating EEEV^FL93^ and VEEV^PIXV^ particles, and (2) discrete lysine residues on the E2 glycoprotein are required to mediate vascular clearance of EEEV^FL93^. While similar, the specific mechanisms mediating the vascular clearance of arthritogenic and encephalitic alphaviruses are distinct, as the vascular clearances of EEEV^FL93^ and VEEV^PIXV^ are independent of MARCO and occur with slower kinetics. Collectively, these findings suggest that phagocytic cells control the magnitude and duration of viremia following infection with a broad group of alphaviruses. Ongoing studies are aimed at defining the specific phagocytic cells and receptors that mediate clearance of circulating EEEV and VEEV^PIXV^ and the molecular features of EEEV and VEEV particles that promote or evade clearance by phagocytic cells. This work was supported by the NIAID of the National Institutes of Health under award number R01 AI148144 awarded to T.E.M. and the National Institutes of Health NIAID training grant (Training Program in Immunology; T32-AI07405) award to S.E.A. All animal studies were performed following guidelines and protocols approved by the Institutional Animal Care and Use Committee of the University of Colorado.

Brendan Monogue^1^, along with Aaron R. Massey^2,3^ (^1^Department of Immunology & Microbiology, ^2^Division of Infectious Diseases, Department of Medicine, University of Colorado Anschutz Medical Campus, ^3^Rocky Mountain Regional VA Medical Center, Aurora, CO, USA) presented their work on how Alpha-synuclein is required for interferon stimulated gene expression in neurons during viral infection. While neurotropic viral infections have been extensively studied, many aspects of the immune responses to such infections in the CNS have yet to be fully explored. Recent studies by the Beckham lab have demonstrated that the neuronal protein alpha-synuclein (α-syn), best known as the causative agent of Parkinson’s Disease, may play a substantial role in activating immune responses during viral infection. α-syn deficient mice have been shown to be substantially more susceptible to infection by West Nile Virus (WNV) and the TC83 strain of Venezuelan Equine Encephalitis Virus (VEEV-TC83) than WT mice. However, the mechanisms of this protection have not yet been explored. Here, they investigate the mechanisms underlying α-syn-dependent immune responses to RNA virus infection in the brain. Using α-syn knock-out (KO) mice and human neuronal models, they show that α-syn is required for expression of the full repertoire of interferon-stimulated genes (ISGs) in neurons following acute RNA virus infection. Furthermore, treatment of α-syn KO human neurons with poly I:C or type I interferon also fail to induce expression of the full complement of ISGs, suggesting that α-syn plays an important role in modulating neuronal innate immune responses. In brain tissue, α-syn-dependent ISG expression is independent of microglia activation and supports activation of infiltrating lymphocytes following viral challenge. These data demonstrate that α-syn is protective against neuroinvasive viruses by facilitating type I IFN responses, demonstrating a unique innate immune response against neuroinvasive viruses within neurons and potentially giving insights into interactions between viral infection and Parkinson’s Disease through this protein’s function. All animal studies were performed following guidelines and protocols approved by the Institutional Animal Care and Use Committee of the University of Colorado Anschutz Medical Campus.

Shiny Queensty (Department of Microbiology, Sri Ramachandra Institute of Higher Education and Research, Porur, Chennai, Tamil Nadu, India) presented her work on atypical manifestations between the association of herpes simplex virus-1 with dementia and inflammatory bowel disease. Herpes simplex virus-1 (HSV-1) enters the gut through virus-laden oral secretions, breaches it and the enteric nervous system; to reach the nodose ganglion. Dormant HSV in trigeminal ganglion undergoes periodic reactivation cycles; spreading to the brainstem, thalamus and sensory cortex; establishing life-long latent infections. Recent evidence suggests that HSV1 reactivation from sensory cortex and nodose ganglion may result in Dementia/Inflammatory Bowel Disease (IBD). The aim of the present study is to determine anti-HSV-1 IgM titres and HSV-1 DNA among participants with dementia and IBD respectively and compare them with the control group. Serum samples from 25 participants with dementia and healthy controls, and colon biopsy samples from 20 IBD and 8 non-IBD participants were collected. Serum samples were analyzed for the presence of anti–HSV-1 IgM antibody titres by ELISA and compared with those of the controls. Biopsy tissue was minced and suspended in Viral Transport Medium and DNA extracted with DNA extraction kit (Qiagen, Germany) by Real-time PCR. Sigmoid curve amplification with the crossing threshold (Ct) value 15–35 was considered positive. Among 25 participants with dementia, 2 had high titres of anti-HSV-1 IgM antibodies. Both were males in their 7th decade of life and had mild/early dementia. Anti-viral therapy was started for one participant who came for follow up. He improved clinically. Out of the 20 participants with IBD, seven (35%) were positive for HSV-1&2 and VZV; (i.e.) 10% (*n* = 2) for HSV-1, 10% (*n* = 2) for HSV-2 and 10% (*n* = 2) for VZV and 1 (5%) for both HSV-1 and VZV. Co-infection with Cytomegalovirus (CMV) was found in two HSV-1 positive participants. Early detection of HSV-1 helps to alert clinicians about the association of HSV–1 with dementia and IBD; enabling them to initiate early therapy in a timely manner thereby benefitting patients. All human studies were performed following protocols and guidelines approved by the Institutional Research Ethics Committee of Sri Ramachandra Institute of Higher Education and Research.

Sarah Stonedahl^1^ along with Penny Clarke^2^, and Kenneth L Tyler^1,2,3^ (^1^Department of Immunology and Microbiology, ^2^Department of Neurology, ^3^Departments of Infectious Disease and Medicine, University of Colorado Anschutz Medical Campus, Aurora, CO, USA) presented their work on the role of microglia in controlling viral growth and subsequent virus-induced mortality in mice infected with West Nile Virus. Encephalitis caused by viral infections is a major cause of hospitalization and death worldwide. West Nile Virus (WNV) is a substantial health concern as it is one of the leading causes of viral encephalitis in the United States today. While most human infections are asymptomatic, a small percentage (~1%) lead to severe neurological disease such as encephalitis. It is estimated that approximately 10% of WNV encephalitis cases result in death, and 50% of patients recovering from neurological disease have long term neurological sequelae. WNV targets neurons in the central nervous system (CNS) and WNV-induced CNS disease in mice is caused, at least in part, by caspase 3-mediated apoptosis. WNV encephalitis in mice is associated with neuroinflammation, including activation of microglia, the resident inflammatory cells of the brain. Depletion of microglia with the CSFR1 inhibitor PLX5622 results in increased WNV titers in the brain and enhanced mortality in a mouse model of WNV-induced CNS disease. They used an ex vivo brain slice culture model to demonstrate that PLX treatment results in increased caspase 3 activation following WNV infection suggesting that microglia are critical in preventing WNV-induced apoptotic neuronal death. Granulocyte macrophage-stimulating colony factor (GM-CSF) increases activation of microglia (and other myeloid cells) and treatment of mice with GM-CSF (Leukine) resulted in increased survival and decreased viral titer in the brain of mice. Taken together these studies provide important insights into the role of microglia during neuroinvasive WNV infections. This research was funded by the National Institute of Health, grant number NS101208 and the Department of Veterans Affairs, grant number BX000963. All animal studies were performed following guidelines and protocols approved by the Institutional Animal Care and Use Committee of the University of Colorado Anschutz Medical Campus.

Amanda Woerman (Department of Biology and Institute for Applied Life Sciences, University Massachusetts Amherst, Amherst, MA, USA) presented her work questioning whether multiple system atrophy is a prion disease. Multiple system atrophy (MSA) is a rare and fatal neurodegenerative disorder characterized by orthostatic hypotension, tremors, rigid muscles, difficulty with posture and balance, impaired speech, and difficulty swallowing. Patients typically develop symptoms in their 50s or 60s and succumb to the effects of autonomic dysfunction within 6–10 years. The brains of MSA patients contain glial cytoplasmic inclusions in oligodendrocytes, which are made of an insoluble and aggregated form of the protein α-synuclein. Over the last five years, they have used cellular and mouse models to investigate the process of α-synuclein misfolding and propagation to better understand the etiology of disease. Through a series of experiments designed to evaluate the pathogenic properties of α-synuclein isolated from MSA patient samples, she determined that misfolded α-synuclein behaves similarly to the misfolded prion protein (PrP^Sc^) responsible for scrapie, chronic wasting disease, and Creutzfeldt–Jakob disease. These findings not only raise important questions about the transmissibility of non-PrP prions but also suggest the need to expand the term prion to include additional misfolded proteins, such as α-synuclein. All animal studies were performed following guidelines and protocols approved by the Institutional Animal Care and Use Committee of the University of California San Francisco.

### 2.4. Session III

David W. Hawman along with Kimberly Meade-White, Shanna Leventhal, Friederike Feldmann, Atsu Okumura, Dana Scott, and Heinz Feldmann (Rocky Mountain Laboratories, NIAID/NIH, Hamilton, MT, USA) presented their work on an immunocompetent mouse model for Crimean-Congo hemorrhagic fever virus. Crimean-Congo hemorrhagic fever (CCHF) is a severe tick-borne febrile illness with wide geographic distribution. CCHF is caused by infection with the Crimean-Congo hemorrhagic fever virus (CCHFV) and case fatality rates can be as high as 30%. Despite causing severe disease in humans, our understanding of the host and viral determinants of CCHFV pathogenesis or disease outcome are limited. A major limitation in the investigation of CCHF has been the lack of suitable small animal models Wild-type mice are resistant to clinical isolates of CCHFV and consequently, mice must be deficient in type I interferon responses to study the more severe aspects of CCHFV. This limits the study of innate responses to the infection, the viral antagonists of these factors and the interaction between innate and adaptive immune responses in the infection. To overcome these limitations, they isolated a mouse-adapted variant of CCHFV that recapitulates in adult, immunocompetent mice the severe CCHF observed in humans. Sequencing identified just 5 coding changes across the viral genome. Infection of wild-type mice with this variant resulted in high viral loads in multiple tissues, inflammatory immune responses and severe pathology of the liver. Unexpectedly, they identified a significant sex-linked bias with female mice largely resistant to severe disease. This resistance was age-dependent and required both innate and adaptive immune responses. Increased disease severity in male mice had similar correlates to more severe disease in humans, including higher viral loads, higher inflammatory immune responses and more severe liver pathology, enabling studies into how these factors contribute to disease outcome. This mouse-adapted variant of CCHFV significantly improves our ability to study host and viral determinants of CCHFV-induced disease in a highly tractable mouse model. This work was funded by the Intramural Research Program of the NIAID, NIH. All animal studies were performed following guidelines and protocols approved by the Institutional Animal Care and Use Committee of Rocky Mountain Laboratories, NIAID/NIH.

Jeffrey M. Grabowski^1^ along with Rosenke R^2^, Long DR^2^, Scott DP^2^, Offerdahl DK^1^, Bloom ME^1^ (^1^Biology of Vector-Borne Viruses Section, Laboratory of Virology, and ^2^Rocky Mountain Veterinary Branch, Rocky Mountain Laboratories, NIAID/NIH, Hamilton, MT, USA) discussed how an *Ixodes scapularis* transcript corresponding to a predicted secreted protein affects flavivirus infection of salivary gland cultures. The ixodid tick vector, *Ixodes scapularis*, transmits several pathogens, including tick-borne flaviviruses (TBFVs). In the US, confirmed human infections with TBFVs deer tick virus (DTV) and Powassan virus (POWV) are increasing and have a fatality rate of 10–15%. In addition, Langat virus (LGTV) is often used as an experimental model TBFV of low neurovirulence. Understanding the functional effect of tick salivary gland transcripts in TBFV infection within *I. scapularis* tick organs is limited and tick ex vivo organ culture is a suitable system to use. In addition, active RNAi response in organ cultures allows research to identify tick genes as possible targets for countermeasure development. Through dsRNA-mediated RNAi transcript knockdown studies, a transcript corresponding to a predicted *I. scapularis* secreted protein (SecP), with in silico predicted lipocalin-like properties, was involved in LGTV and DTV infection in salivary gland (SG) cultures from unfed female ticks. SecP is suggested to be proviral, as transfection of dsRNA corresponding to SecP resulted in reduced infectious LGTV and DTV replication without affecting SG viability. In ongoing work, localization of SecP transcript in SGs is being explored. DsRNA-mediated RNAi transcript knockdown studies with SecP in *I. scapularis* ticks is underway, including evaluation of transcript knockdown of SecP on TBFV infection of ticks, tick attachment, and blood uptake. Additionally, the effect of SecP protein (has in silico-predicted antigenicity) and corresponding antibody on TBFV infection, tick attachment, and blood uptake will provide valuable insight. This research was supported by the Intramural Research Program of the NIH, NIAID. No animal or human studies were performed.

Frances S. Li, along with Kathryn S. Carpentier and Thomas E. Morrison (Department of Immunology and Microbiology, University of Colorado School of Medicine, Aurora, CO, USA) discussed surface features of chikungunya virus (CHIKV) particles that are important for viral clearance from circulation. Arboviruses, such as mosquito-borne alphaviruses, are major public health concerns, and the capacity of an arbovirus to be transmitted in a human-mosquito-human transmission cycle has fueled disease outbreaks worldwide. Major determinants of arbovirus transmission, geographic spread and pathogenesis are the magnitude and duration of viremia in the vertebrate host. Previously, they found that multiple alphaviruses, including CHIKV and Ross River (RRV) viruses, are cleared efficiently from the circulation of mice by liver Kupffer cells and the cell surface scavenger receptor A6 (MARCO). MARCO-dependent clearance of these viruses was contingent on the presence of a lysine (K) residue at position 200 of the E2 glycoprotein (E2 K200) in CHIKV or E2 K251 in RRV. Substitution of these K residues, regardless of conservative or radical, abrogated clearance of circulating viral particles and resulted in enhanced viral dissemination. To determine whether other exposed residues in the CHIKV E2-E1 trimeric spike are important for MARCO-dependent clearance, they performed structure-guided alanine substitutions at additional residues surrounding the E2 K200 site. This analysis revealed that E2 E208 and E1 K61 also are required for efficient clearance of circulating CHIKV particles in vivo. To assess whether the charge property of E2 E208 or E1 K61 is important for viral clearance, they introduced the following amino acid substitutions: E208D/R and K61E/R/H. Evaluation of these particles in mice revealed that E2 E208D and E1 K61R/H variants were cleared from circulation as efficiently as WT CHIKV, whereas E2 E208R or E1 K61E CHIKV mutants escaped MARCO-dependent clearance. These results suggest that there are distinct charge distributions surrounding E2 K200 that may facilitate ionic interactions between CHIKV particles and the scavenger receptor cysteine rich domain of MARCO, and they plan to assess whether similar charge requirements exist surrounding E2 K251 of RRV for MARCO-dependent viral clearance from circulation. This work is supported by the following funding sources: AI123348, AI148144, and AI140567. All mice experiments were performed at the University of Colorado, Anschutz Medical Campus and adhere to the Institutional Animal Care and Use Committee (IACUC) guidelines.

Jillian N. Whelan^1^ along with Joshua Hatterschide^2^, Erick R. Perez^1^, Elizabeth A. White^2^, and Susan R. Weiss^1^ (^1^Department of Microbiology, ^2^Department of Otorhinolaryngology: Head and Neck Surgery, Perelman School of Medicine, University of Pennsylvania, Philadelphia, PA, USA) presented their research on organotypic skin cultures as a three-dimensional human skin model for flavivirus infection. Flavivirus-infected mosquitoes deposit virus into the dermis while taking a blood meal; therefore, determination of the initial host–virus interactions enabling infection in the skin can facilitate development of antivirals targeting this primary infection site. However, a genetically tractable human skin model for studying RNA viruses has yet to be developed. They constructed 3D organotypic epithelial cultures as a model for evaluating host-flavivirus interactions crucial to infection and spread in the skin. Organotypic cultures, comprised of a stratified squamous epithelium of primary human keratinocytes and a dermal layer of primary human fibroblasts, were infected with Zika, dengue, or West Nile viruses. They demonstrated proper generation of dermal and epidermal layers by H&E staining and immunofluorescence (IFA) staining. They used IFA and fluorescence in situ hybridization (FISH) staining for viral protein and RNA to identify specific cell types targeted by each virus, and confirm organotypic cultures as a suitable model for flavivirus skin infection. Additionally, they used IFA staining to evaluate changes in host innate immune, inflammatory, and cell death responses in these cultures as a result of flavivirus infection. As cells comprising organotypic raft cultures are amenable to gene editing, their current efforts towards further advancing this model include CRISPR-Cas9-mediated knockout (KO) of specific host genes in primary skin cells prior to construction of 3D cultures, as well as the incorporation of Langerhans cells and dermal dendritic cells into the epidermal and dermal layers of the organotypic epithelial cultures. No animal or human studies were performed.

### 2.5. Session IV

Rebecca Broeckel (Laboratory of Virology, Rocky Mountain Laboratories, NIAID, Hamilton, MT, USA) shared her work on a genome-wide CRISPRa screen that revealed helicase with zinc finger 2 as a potent cellular inhibitor of Ebola virus. Ebola virus (EBOV) causes outbreaks of viral hemorrhagic fever in humans with a case fatality rate of 60%. EBOV is a major public health concern because of its extreme virulence, its capacity for human-to-human transmission, and the unpredictability of EBOV emergence. The host type I interferon (IFN) response is critical in controlling EBOV replication at the cellular level, but the specific host proteins that have direct antiviral activity against EBOV, termed restriction factors, are unknown. To identify new host restriction factors for EBOV, they employed a genome-wide CRISPR transcriptional activation (CRISPRa) screen in human liver cells. In this CRISPRa screen, endogenous gene expression is induced by a nuclease-dead Cas9, recruited transcriptional activators, and guide RNAs that target promoter regions (10 guides/gene, ~210,000 guides in total). To identify putative viral inhibitors, CRISPRa cells were infected with EBOV whose replication is cytopathic, and surviving cells were subjected to RNAseq. After selection with EBOV, approximately 300 genes were significantly enriched in surviving cells. The top antiviral gene candidate was helicase with zinc finger 2 (Helz2), which is a known IFN-stimulated gene. Helz2 proved to be a remarkably potent restriction factor for EBOV by blocking release of infectious virus by more than 1000-fold compared to the control. To identify the mechanism of Helz2 inhibition, viral RNA was measured over a single round of infection during Helz2 overexpression. Helz2 interfered with EBOV RNA replication, while virus binding and initial entry into cells were not affected. This work identifies a specific point of vulnerability of EBOV during RNA replication and thereby reveals a strategy to design antivirals that mimic Helz2 function. Validation of additional ‘hits’ from this screen will expand our understanding of the antiviral gene repertoire of the cell against EBOV. This work was funded by the Intramural research Program of the NIAID. No animal or human studies were performed.

Chaoping Chen (Department of Biochemistry and Molecular Biology, Colorado State University, Fort Collins, CO, USA) presented her research on trans proteolysis of HIV-1 protease precursors independent of their dimerization propensity. HIV-1 protease (PR) is temporospatially regulated to suppress mature protease liberation from its Gag-Pol polyprotein precursor until virion release. Their lab previously established a cell-based assay to study autoprocessing of precursors carrying the PR plus its N-terminal sequence (i.e., p6*-PR miniprecursor) sandwiched between various flanking tags. This report specifically examined precursor *trans* proteolysis inside mammalian cells under conditions where precursor autoprocessing is prevented. Positive *trans* proteolysis was detected by constructs carrying dimer-forming GST, or monomeric C2-MBP, or even short peptides upstream of the p6*-PR miniprecursor, indicating that *trans* proteolysis is independent of a dimer-inducing tag. However, no detectable *trans* proteolysis was observed when lysates of cells individually expressing the enzyme and substrate were mixed in vitro, indicating requirement of other factors in this process. Sucrose gradient sedimentation analysis revealed dimerization and oligomerization of substrate precursors as an intrinsic property that was insensitive to RNase A and nonionic detergent treatment. Immunoprecipitation identified complexes containing both enzyme and substrate precursors with or without a dimer-inducing tag. The dimerization propensity of an enzyme precursor varied under different contexts, demonstrating its conformational flexibility in response to variations within and beyond the p6*-PR coding region. Additionally, the p6*-PR precursors, as well as p17 (MA) and p24 (CA), were mostly monomeric when the viral particles released from transfected cells were solubilized with non-ionic detergents and subjected to sucrose gradient sedimentation analysis. Collectively, her results decoupled *trans* proteolysis activity from the requirement for dimer-inducing sequences upstream of p6*-PR and demonstrated conformational flexibility involved in protease autoprocessing. This work was supported by the National Institutes of Health grants 1R01AI50223 and 1R01AI120365. No animal or human studies were performed.

Henry Dunkleberger (Microbiology, Immunology, Pathology Department, Colorado State University, Fort Collins, CO, USA) presented his work on the identification of endogenous feline leukemia virus (enFeLV) long terminal repeat (LTR) integration sites in three populations of domestic cats of varying genetic diversity. While endogenous retroviruses (ERV) offer us a unique perspective of our evolutionary history, they also play important parts as homeostatic regulators. Though ERVs no longer encode for infectious virus, their long terminal repeats (LTRs) act as promoters for *cis*-activation of host genes and enhancer elements for *trans*-activation of host genes up to 1Mb from their insertion site. Endogenous murine leukemia virus LTRs integrated proximal to host anti-viral genes promote the expression of proteins that restrict against exogenous retroviral infections. His lab has previously documented a negative dose-dependent correlation between endogenous feline leukemia virus (enFeLV) LTR and exogenous infection, suggesting enFeLV-LTR-mediated viral restriction. They, then, therefore assessed enFeLV-LTR integration site diversity and examined genes in close proximity to enFeLV-LTR integration sites that could act as anti-viral genes. Using a targeted linker-mediated PCR approach with deep sequencing, they identified LTR integration sites in 20 domestic cats from three populations with varying degrees of inbreeding. Reads were mapped to a domestic cat reference genome to assess. LTR integration sites were manually confirmed and BLASTed the mapped sequences to confirm identity and compiled all LTR integration cites per population. They evaluated the most commonly shared LTR integration sites among individuals, as well as LTRs that were unique to individuals. Their results indicate that while total number of integration sites did not differ between populations, outbred animals had a greater number of unique integration sites. One of the gene families identified proximal to LTR integration sites were zinc finger proteins that have a variety of regulatory functions. This study identified a new technology to assess ERV integration sites in individual animals, associate similarity in integration sites with degree of relatedness, providing a baseline for inquiry into endogenous retroviral control of host genes in the domestic cat. Funding for this project was provided from the following sources: NSF (EID 1413925), NIH (F30 OD023386), and Winn Feline Foundation Feline New Investigator Award in Genomics (W18-013). All animal studies were performed following guidelines and protocols approved by the Institutional Animal Care and Use Committee of Colorado State University.

Ryan H Jeep along with Christian L Topete, Liangqun Huang, and Chaoping Chen (Department of Biochemistry and Molecular Biology, Colorado State University, Fort Collins, CO, USA) addressed diverse catalysis activities manifested by HIV-1 protease inhibitor resistance mutants in his talk. HIV protease inhibitor (PI) resistance is an ongoing problem that compromises treatment efficacy and prognosis of combination antiretroviral therapy (cART). While extensive sequence analysis has identified numerous major and minor resistance-associated mutations (RAMs) in the protease (PR) gene, how these mutations confer PI resistance is not fully understood. He reported here the development of an assay that allows examination of HIV mature and precursor proteases in transfected mammalian cells. He engineered an expression vector to directly produce the mature PR or precursor mediated by the P2A peptide derived from porcine teschovirus-1 via highly efficient ribosomal skipping. The resulting mature or precursor proteases underwent self-degradation and were also competent at trans-proteolysis of substrates co-expressed in cells, both of which can be suppressed by HIV-1 protease inhibitors. He examined a panel of mutants that were previously identified in patients experiencing drug resistance. Self-degradation of some mature PRs was highly resistant to suppression by protease inhibitors, which was consistent with reported drug resistance. Different mature PRs demonstrated various trans-proteolysis efficiencies on the same substrate. Moreover, precursor and mature PRs responded to PI treatment differently showing different IC_50_ values. Collectively, their results illustrated diverse catalysis activities associated with various mutants, suggesting multiple and complicated mechanisms involved in drug resistance. This work was supported by the National Institutes of Health grants 1R01AI50223 and 1R01AI120365. No animal or human studies were performed.

### 2.6. Session V

Claire Birkenheuer along with Joel Baines (Pathobiological Sciences Department, School of Veterinary Medicine, Louisiana State University, Baton Rouge, LA, USA) presented their work on how HSV-1 ICP22 is required to maintain cellular RNA Polymerase II on the viral genome during lytic infection. During lytic herpes simplex virus-1 (HSV-1) infection, 60% of the host transcriptional machinery is redirected to the viral genome within 6 h post infection (hpi). The ICP22 protein, one of four immediate early HSV-1 proteins that modifies the cellular transcriptional machinery, inhibits phosphorylation of the cellular RNA polymerase II (Pol II) carboxyl terminal domain (CTD). They performed precision nuclear run (PRO-seq) on Hep2 cells infected with an ICP22 deletion (ICP22del) virus at 3 and 6 hpi and compared it to infection with a virus bearing a restored ICP22 encoding gene. The nucleotide-resolution snapshot of the location of active Pol II under these conditions showed that ICP22 is required to maintain Pol II on the viral genome. At 3 hpi, the level of Pol II on the viral genome was similar in cells infected with the two viruses (~85 and ~129 active Pol II molecules/1000 nucleotides (APM/1000NT) of viral genome in the ICP22del and repaired virus infections, respectively). At 6 hpi this number increased to ~500 APM/1000NT on the repaired viral genome but decreased to 56 APM/1000NT on the ICP22del viral genome. Moreover, Pol II returned to the human genome in cells infected with the ICP22del infection, with 90% of the host genes regaining Pol II from 3 to 6 hpi in the deletion virus infection. Current efforts are designed to determine the mechanism ICP22 uses to retain Pol II on the viral genome. No animal or human studies were performed.

Erin Borland along with Nicholas A. Bergren, Daniel Hartman, and Rebekah Kading (Department of Microbiology, Immunology and Pathology, Colorado State University, Fort Collins, CO, USA) presented an update on their laboratory demonstration of transovarial transmission of Rift Valley fever virus in *Culex tarsalis* mosquitoes. Rift Valley fever virus (RVFV) is an emerging mosquito-transmitted virus with a demonstrated ability to invade previously naïve geographic areas. Current dogma, based on limited field evidence, is supportive of RVFV being maintained by transovarial transmission (TOT) from parent mosquito to offspring. Understanding the vertical transmission dynamics of RVFV is crucial for determining the risk of RVFV establishment in mosquito populations of the United States. However, demonstration of vertical transmission of RVFV in the laboratory by any mosquito vector species is lacking. To address this important knowledge gap, they orally challenged *Culex tarsalis* mosquitoes (KNWR strain) with an epidemic strain of RVFV from Kenya and tracked infection rates in progeny over three consecutive gonotrophic cycles. Progeny from all three gonotrophic cycles were reared to adults, with representatives from each developmental stage assayed for the presence of infectious virus by plaque assay. The infection and transmission rates among the parental generation were 72.0% and 40%, respectively. Infectious virus was recovered from ovarian tissues of parental mosquitoes after the first infectious blood meal. Infection via TOT was confirmed in progeny after the first ovarian cycle, with infection rates of F1 adult mosquitoes from different gonotrophic cycles ranging from 2.0–10.0%. Infectious virus was also recovered from the ovaries and salivary glands of adult F1 progeny. These data confirm that RVFV can be passed transovarially in *Cx. tarsalis* mosquitoes. The relative contribution of TOT for promoting the establishment of RVFV in native mosquito populations in the United States is unknown. No animal or human studies were performed.

Daniel A. Hartman (Arthropod-borne & Infectious Diseases Laboratory, Department of Microbiology, Immunology, and Pathology, Colorado State University, Fort Collins, CO, USA) presented an update about vector competence and vertical transmission potential of wild caught mosquitoes for Rift Valley Fever Virus in Colorado. Rift Valley fever virus (RVFV) is a mosquito-borne pathogen that infects ruminants and humans. In ruminants, RVFV causes spontaneous abortions and is nearly 100% fatal in young animals; in humans, the virus can cause febrile illness with some cases of blindness, hemorrhagic fever, and mortality. RVFV is maintained in nature, in part, by vertical transmission among floodwater *Aedes* spp. mosquitoes. The increase in RVFV outbreaks outside of its historical endemic range of Africa is worrisome, indicating major threat for introduction to North America. Recent work has demonstrated very high susceptibility of white-tailed deer to the virus, suggesting the ability of RVFV to establish in North American wildlife. While many mosquito species have demonstrated the ability to transmit RVFV under laboratory conditions, data for some Colorado species are lacking, including species known to feed on deer. They performed experiments to assess vector competence of local mosquito species, utilizing field-collected *Aedes melanimon*, *Aedes increpitus*, and *Culiseta inornata.* Field-collected *Culex tarsalis* and *Aedes vexans* were included to confirm previous vector competence data, while describing rates of infection, dissemination, transmission, and vertical transmission potential. *Aedes melanimon*, *Aedes vexans*, and *Aedes increpitus* showed high rates of infection, but little viral dissemination, indicating midgut escape barriers. *Culex tarsalis* showed high transmission rates as previously reported, as did *Culiseta inornata*. Virus was detected in the ovaries *Aedes melanimon*, *Aedes vexans*, and *Culiseta inornata*, indicating potential for these species to transmit RVFV vertically. These data show the possibility for multiple modes of RVFV transmission by mosquitoes among wild deer hosts in Colorado. No animal or human studies were performed.

Ashley Knox^1^ along with Eva Medina^1^, Rachel Erickson^1^, Darby Oldenburg^2^, and Linda van Dyk^1^ (^1^Department of Immunology and Microbiology, University of Colorado Anschutz Medical Campus, Aurora, CO, USA; ^2^Gundersen Medical Foundation, La Crosse, WI, USA). presented their work on conserved and sequence-independent functions of gammaherpesvirus non-coding RNAs. Gammaherpesviruses (γHVs) have evolved with their hosts for millions of years and, as such, have become experts in modulating host cells to mediate lifelong infection. One method of achieving this is through the use of non-coding (nc) RNAs. The γHV ncRNAs, including γHV68 TMERs and Epstein-Barr virus EBERs, are abundantly expressed and required for pathogenesis. The γHV68 genome contains eight TMERs, each with tRNA-like structures followed by stem loops that are processed into miRNAs. Previous work in their lab showed that knock-out of all TMER genes abrogated acute pulmonary inflammation; however, a single TMER (TMER1) was able to restore pathogenesis, even without the associated miRNAs. These data suggest a sequence-independent function of the TMERs. Considering the similarity in structure across various TMERs, they hypothesized that this function was not unique to TMER1 and that other individual TMERs could restore pathogenesis. To test this, they generated multiple single-TMER virus recombinants that express either TMER4, TMER5, or TMER8, as well as a viral recombinant that expresses the EBV EBERs in place of the TMERs. They measured acute pathogenesis and virus replication of these recombinants in infected lungs. They found no significant differences in virus replication, but found that expression of any single TMER or the EBERs is sufficient to restore pathogenesis to wild-type virus levels, suggesting functional redundancy across these virus ncRNAs. In addition to the shared, sequence-independent role of the virus ncRNAs in vivo, they showed that the TMERs and EBERs share the ability to bind to the RNA binding proteins, RIG-I and La/SSB. Current work is focused on identifying the population of ncRNAs that interact with these proteins during infection and how these interactions alter acute responses and pathogenesis. These studies reveal the conserved, sequence-independent role of γHV ncRNAs in their interactions with the host. This work was supported by the NIH grants R01AI121300 to LFvD, T32 AI052066 to ANK and CO RNA Biosciences summer internship support to RE. All animal studies were performed following guidelines and protocols approved by the Institutional Animal Care and Use Committee of the University of Colorado Anschutz Medical Campus.

Rosemary Rochford (Department of Immunology and Microbiology, University of Colorado, Anschutz Medical Campus, Aurora, CO, USA) presented her talk on a tale of two strains and uncovering the T-cell Tropism of EBV Type 2. Epstein-Barr virus (EBV) has a well-described tropism for B-cells and epithelial cells. Most studies on EBV tropism have been performed using the predominant strain of EBV, EBV Type 1. However, there are two strains of EBV, Type 1 and 2. EBV Type 2, similar to EBV Type 1, can infect B-cells. However, they have also found that EBV Type 2 has an additional tropism for CD3+ T-cells. In this talk, she reviewed their studies on the infection of T-cells by EBV Type 2 both in vitro and in vivo, the viral and cellular receptors for entry into T-cells and consequences of T-cell infection. The T-cell tropism of EBV Type 2 has implications for EBV vaccines and also implications for disease pathogenesis of EBV+ T cell malignancies. All animal studies were performed following guidelines and protocols approved by the Institutional Animal Care and Use Committee of the University of Colorado, School of Medicine. All human studies were approved by COMIRB.

### 2.7. Session VI

Brianna Bullard along with Brigette Corder, and Eric Weaver (School of Biological Sciences, Nebraska Center for Virology, University of Nebraska, Lincoln, NE, USA) presented her research on how an epigraph hemagglutinin vaccine induces broad cross-reactive immunity against swine H3 Influenza Virus. Influenza A virus in swine (IAV-S) is a significant burden on the pork industry and a threat to human health due to its zoonotic potential. Since 2010, there have been >450 swine influenza variant infections in humans, with the majority of those cases serotyped as H3N2v. Current IAV-S vaccines only induce strain-specific immunity and often do not match the circulating strain. Here, they explored the use of the epigraph vaccine designer tool for the production of a universal swine H3 (swH3) influenza vaccine. This tool uses a graph-based computational algorithm to design a cocktail of three hemagglutinin (HA) immunogens that maximize the potential epitope coverage of the diverse swH3 population. These epigraph immunogens were delivered using an Adenovirus type 5 vector and compared to a wildtype HA or the commercial inactivated IAV-S vaccine, FluSure. Epigraph immunized mice developed protective antibody levels against 14 of the 20 (70%) divergent swH3 strains tested, whereas the wildtype HA and FluSure vaccines only induced protective antibody levels against 3 of the 30 (15%) and 4 of the 20 (20%) strains, respectively. The epigraph vaccine also induced a stronger T-cell response and recognized a greater number of epitopes against four divergent swH3 strains. Challenge studies with three divergent swH3 showed that mice vaccinated with the epigraph vaccine had reduced weight loss and lung viral titers as compared to the wild type HA and FluSure. Finally, a vaccine study in outbred swine, the target species for their vaccine, showed that the epigraph vaccine induced superior levels of immunity with stronger antibody development, greater cross-reactive antibodies, and a stronger total T cell response as compared to the other vaccines. These data support the development of an epigraph vaccine as a universal swH3 vaccine capable of providing cross-protection against highly divergent strains of swH3. This research was supported by the National Institutes of Health under Ruth L. Kirschstein National Research Service Award 1 T32 AI125207. All animal studies were performed following guidelines and protocols approved by the Institutional Animal Care and Use Committee of the University of Nebraska—Lincoln.

Bridgette Corder along with Bullard B, Hoagstrom K, Jahnke L, and Eric Weaver (School of Biological Sciences, Nebraska Center for Virology, University of Nebraska-Lincoln, Lincoln, NE, USA) presented an update on improved immunogen design for influenza vaccines using an epigraph hemagglutinin influenza B vaccine. Influenza viruses cause 3–5 million cases globally. Despite the vast majority of research focusing on influenza A virus, influenza B virus (IBV) is responsible for most pediatric and young adult influenza cases reported each year. The main method of protection against influenza is vaccination but current influenza vaccines only provide transient, strain-specific immunity. There is an urgent need for vaccines that induce cross-protective immunity against the broad diversity of influenza. Their lab has developed and tested a novel hexavalent hemagglutinin (HA) epigraph vaccine against human IBV. This vaccine includes three immunogens for both main IBV populations, Yamagata and Victoria. Epigraph immunogens are optimized in silico to include the most potential B and T cell epitopes (PBTE) from a diverse population. Phylogenetic analysis confirms that the epigraph HAs provide broad coverage of the diverse IBV strains. Each epigraph immunogen was expressed in recombinant Adenovirus type 5 for analysis in mice and compared to a commercial inactivated vaccine, Fluzone. They will continue to analyze the cellular and humoral immunity induced by vaccination. Additionally, vaccine efficacy will be determined by weight loss and death after lethal IBV challenges. Preliminary data show that, when mismatched, the epigraph vaccine induces broader immunity than seasonal influenza vaccines. This study will clarify our understanding of optimized immunogens for diverse viral populations. This project was funded by the National Institutes of Health Ruth L. Kirschstein National Research Service Award 1 T32 AI125207. All animal studies were performed following guidelines and protocols approved by the Institutional Animal Care and Use Committee of the University of Nebraska—Lincoln.

Carley McAlister^1^ along with Elena Lian^1^, Gabriela Ramirez^1^, Loran Anderson^1^, Laura St Clair^1^, Justin Hoopes^2^, Brian Geiss^1^, and Rushika Perera^1^ (^1^Microbiology, Immunology, Pathology Department, Colorado State University, Fort Collins, CO, USA; ^2^AVR Laboratories, Logan, UT, USA) presented an update regarding COVID-19 antiviral testing efforts at Colorado State University. COVID-19 displays unexpected complications that require unique approaches to therapeutic intervention. Increasing evidence suggests that while monotherapies of FDA approved drugs may have some ameliorating effects they fall short to provide effective intervention. This is primarily due to the unique metabolic interactions in individuals that exacerbate variability to the drug’s response causing idiosyncratic, hyporeactive, hyperreactive or tachyphylaxis manifestations. Two innovative approaches will provide a significant leap forward to improve therapeutic efficacy; (i) Identifying synergistic combination therapies that will provide the ability to utilize low-dose interventions that have enhanced efficacy and higher therapeutic indices through synergy.

Michael J. Rudy (Department of Neurology, University of Colorado School of Medicine, Aurora, CO, USA), discussed the association of enterovirus D68 particles with exosomes. Enterovirus D68 (EV-D68) is an emerging pathogen which causes respiratory disease and is associated with a poliomyelitis-like syndrome in children. The incidence of disease caused by EV-D68 have been increasing but factors influencing its emergence remain largely unknown. Here, he described three distinct densities of infectious virus that were identified from density gradient centrifugation of EV-D68 laden supernatant from infected cells. Electron microscopy revealed that the lowest density peak (centered at 1.11 g/cm^3^) was associated with what appeared to be membranous vesicles. Treatment with detergent prior to centrifugation, shifted both infectivity and genome copy number to the higher density peaks (at 1.20 g/cm^3^ and 1.24 g/cm^3^), confirming viral association with lipid membranes. Further experiments using anti-EV-D68 antibodies indicated that the membrane does not protect virus from immune recognition and that naked viral particles are attaching to the exterior surface of membranous vesicles. Further characterization of the membranous vesicles revealed that they express CD81 and CD63, two tetraspanins that are exosome specific. Taken together, he showed that EV-D68 naked viral particles (at 1.20 g/cm^3^ or 1.24 g/cm^3^) can adsorb to the exterior surface of exosomes to generate this lowest density fraction (1.11 g/cm^3^). Furthermore, the viral particles that equilibrated at 1.20 g/cm^3^ density were approximately 20× more infectious in contemporary EV-D68 isolates as compared to the prototypic Fermon strain, highlighting differences between contemporary and ancestral virus. Funding for these studies was provided by NIH grants R01 NS101208 and F30 AI36403-01A1. No animal or human studies were performed.

Laura A. St Clair (Center for Vector-borne Infectious Diseases; Microbiology, Immunology, Pathology Department, Colorado State University, Fort Collins, CO, USA) presented her work on the role of human sialidases during flavivirus infection. The human sialidase enzymes (or neuraminidases) catalyze the removal of α-glycosidically linked sialic acid residues from glycoconjugates, including glycoproteins and glycolipids. Sialic acid residues are thought to play vital roles in cellular signaling. Through their physiochemical effect on glycoconjugates, sialic acid residues are involved in conformational changes that determine the active/inactive state of glycoproteins. Sialic acid residues are also important binding recognition and masking sites for cellular processes. There are four known human neuraminidases (NEU1-4), each having specific subcellular localizations and substrate preferences. In previous studies, NEU1-4 activity has been shown to be increased during infection with dengue virus serotype 2 (DENV2). In both in vitro and in vivo models, it was shown that the dengue NS1 protein increased NEU1-4 activity, resulting in an increase in free sialic acid. This increased activity was also shown to be linked to the events resulting in endothelial hyperpermeability/vascular leakage, a hallmark of severe dengue disease. However, the reason for this increase in NEU1-4 activity was not understood. In an siRNA screen of enzymes involved in the sphingolipid metabolic pathway, Laura uncovered that NEU1-4 are vital for DENV2 infectious virus release and replication. Here, she presented data shedding light on the roles of NEU1-4 during the DENV lifecycle. No animal or human studies were performed.

Sydney J. Bennett (Nebraska Center for Virology and School of Biological Sciences, University of Nebraska-Lincoln, Lincoln, NE, USA) presented her work on the generation of human monoclonal antibodies against Kaposi’s Sarcoma Herpesvirus. To generate human monoclonal antibodies against Kaposi’s Sarcoma Herpesvirus (KSHV) that can be used to characterize host immune responses, for studies that focus on treatment or vaccine development, and as useful reagents for characterization of KSHV infection, she single-cell sorted CD20+CD27+ memory B cells from 3 Kaposi’s Sarcoma (KS) patients resulting in 1117 individual memory B cells. From the single-cell cDNA, she successfully amplified heavy chain variable regions from 509/1117 wells (43%) and from those both kappa and lambda light chains were amplified resulting in 465/509 light chains—299 kappa, 187 lambda, and 21 double positive. Importantly, she has matched heavy and light chain variable regions of 461/1117 (41.3%) antibodies. The variable regions were sequenced and aligned with NCBI IgBLAST to define the allotype, ensure they have a productive open reading frame, and determine the correct cloning primers to use to prepare the amplified variables regions for Gibson Assembly. She is currently working through the cloning PCR to add the Gibson Assembly overhangs, followed by the Gibson Assembly reaction and transformation into competent cells. For a select number of antibody clones, she has demonstrated antibody expression by co-transfecting the heavy and light chain plasmids into HEK293A, followed by harvesting the antibody-containing supernatant. Protein lysates were analyzed on SDS-PAGE followed by Western blot with anti-human IgG. After Western blot confirmation, the antibodies are being tested for KSHV-specificity by monoclonal-enhanced immunofluorescence assay (mIFA). Thus far, she has identified two antibodies that bind KSHV infected cells by mIFA. Further characterization of these antibodies is ongoing. Funding for this project was provided by NIH U54 CA221204, NIH U54 CA190155, and NIH P20GM103427. All studies using human subjects or tissue samples have been either approved or deemed non-human subject research by the Institutional Review Board of the University of Nebraska-Lincoln.

